# Use and diagnostic outcomes of cancer patient pathways in Denmark – is the place of initial diagnostic work-up an important factor?

**DOI:** 10.1186/s12913-022-07545-x

**Published:** 2022-01-31

**Authors:** Christina Sadolin Damhus, Volkert Siersma, Anna Rubach Birkmose, Susanne Oksbjerg Dalton, John Brodersen

**Affiliations:** 1 The Research Unit for General Practice and Section of General Practice, Department of Public Health, University of Copenhagen, Øster Farimagsgade 5, 1014 Copenhagen, Denmark; 2 Primary & eHealth Care, Region Zealand, Alléen 15, 4180 Sorø, Denmark; 3 Survivorship & Inequality in Cancer, the Danish Cancer Society Research Center, Strandboulevarden 49, 2100 Copenhagen, Denmark; 4 Department of Clinical Oncology & Palliative Care, Zealand University Hospital, Ringstedgade 61, 4700 Næstved, Denmark

**Keywords:** Cancer patient pathways, Cancer diagnostics, Prognostic outcomes, Registry-study

## Abstract

**Introduction:**

The Cancer Patient Pathway for Non-specific Symptoms and Signs of Cancer (NSSC-CPP) has been implemented in Denmark with regional and intra-regional differences. In some places, the initial diagnostic work-up (often including a CT scan) is performed by general practitioners (GPs) and in others by hospitals. Variations may influence the use of Organ Specific Cancer Patient Pathways (OS-CPPs) and prognostic outcomes for the patients. Therefore, the aims were: 1) To analyse how a CT scan referred from GP or hospital is followed by OS-CPPs and NSSC-CPPs at the national and regional level, and 2) To analyse, nationally and regionally, the diagnostic outcomes of persons referred to CT scan by either GP or hospital six months after and mortality one year after CT scan.

**Methods:**

A nationwide population-based study including individuals with a first CT scan in 2013-2016, either referred from GP or hospital.

**Results:**

Overall, individuals with a CT scan referred from GPs were more likely to start a NSSC-CPP or an OS-CPP than individuals with a CT scan referred by hospitals. Across the five Regions in Denmark, CT scans referred by GPs were associated with reduced odds of total mortality in all regions; (North, OR=0.78 [0.73 0.83], Central, OR=0.92 [0.87 0.96], South, OR=0.85 [0.81 0.89], Capital, OR=0.96 [0.91 1.00] and Zealand, OR=0.85 [0.79 0.90]) and increased odds of cancer-specific mortality in four regions, ORs ranging from 1.15-1.51 with no difference in Region North (1.00 [0.91 1.10]).

**Conclusion:**

No obvious association between more CT scans and CPPs and reduced diagnoses and mortality was observed. The different diagnostic models might not explain the prognostic outcomes, but the different use of CT scans in, and between Regions play a large role in the differences in incidence and mortality.

**Supplementary Information:**

The online version contains supplementary material available at 10.1186/s12913-022-07545-x.

## Background

In 2008, as the first country in the world, Denmark implemented Cancer Patient Pathways (CPPs) which are organised pathways including diagnostic work-up, treatment and follow-up care [1]. The aim was to ensure well-planned pathways to improve the prognosis and the quality of life for patients. By 2021, 33 organ-specific CPPs (OS-CPPs) have been implemented. The introduction of CPPs has reduced waiting time for cancer treatment [2], but the prognostic benefits of implementing CPPs have been discussed [2–4]. The OS-CPPs are initiated based on specific alarm symptoms (such as rectal bleeding, dysphagia or a lump) relating to suspicion of cancer in a specific organ (colorectal, esophagus or breast cancer, respectively). Specific guidelines for referral criteria, time consumption and steps in the diagnostic work-up have been developed for each OS-CPP [5]. With inspiration from the Danish model, CPPs have since been introduced into the Swedish [6–9], Norwegian [10] and UK healthcare system[11–13].

In patients with cancer, 50% present with either vague or non-specific symptoms, (e.g. unexplained weight loss, pain or fatigue) which was the rationale for implementing the national health strategy – *‘The sooner the better’* where one of the main elements was easier access for general practitioners (GPs) to refer to diagnostic imaging at the hospital [14–16]. Further, the CPP for non-specific symptoms and signs of cancer (NSSC-CPP) was implemented [14, 15]. GPs have a substantial role in the initial diagnostic work-up of these patients, as they have the challenging role of filtering the few patients with cancer among the many [17]. To initiate a NSSC-CPP, the GP is expected to order a specific blood panel and if necessary an X-ray or CT scan (conducted in secondary care facilities) to rule out the suspicion of cancer or other serious disease [18]. If the GP’s suspicion remains after the initial testing, the GP is advised to refer the patient to a diagnostic unit at hospital. However, due to different regional and intraregional implementations of the NSSC-CPP in Denmark, some GPs can refer patients to the diagnostic units without the initial diagnostic work-up [19]. In a previous study, we found that in one of the five Regions in Denmark, the GPs were responsible for the initial diagnostic testing, and in another it was the diagnostic unit. In the remaining three Regions, there were intraregional differences in the set-up of the NSSC-CPP [19]. The NSSC-CPP is registered when the diagnostic unit accept the referral, while the potential initial blood panel and CT scan managed in general practice are not. Therefore, the registered NSSC-CPPs do not include the same initial procedures across the country, which challenge measuring the outcomes of the NSSC-CPPs and thereby evaluating their quality. At the moment, the Danish Health Authorities measures the quality of the NSSC-CPP based on the number of completed NSSC-CPPs and within recommend time frames without including patient-relevant prognostic outcomes. One study indicates that the route to diagnosis is associated with the prognosis of patients with cancer [20]. Still, no studies have examined if the regional/intraregional organisation of the initial diagnostic work up affects the use of CPPs. Also, current Danish studies regarding the diagnostic outcomes and mortality following NSSC-CPPs are based on local and not on national data [21–28]. Therefore, the aims of this study were two-fold: 1) To analyse how a CT scan referred from GP or hospital is followed by OS-CPPs and NSSC-CPPs at the national and regional level, and 2) To analyse, nationally and regionally, the diagnostic outcomes of these two CT-groups six months and mortality one year after CT scan.

## Material and methods

### Study design

This study was a nationwide population-based observational cohort study on individual-level register data, obtained by linking Danish national registers using the unique personal identity number assigned to all Danish citizens at birth or immigration [29].

### Study population

**Fig. 1:**
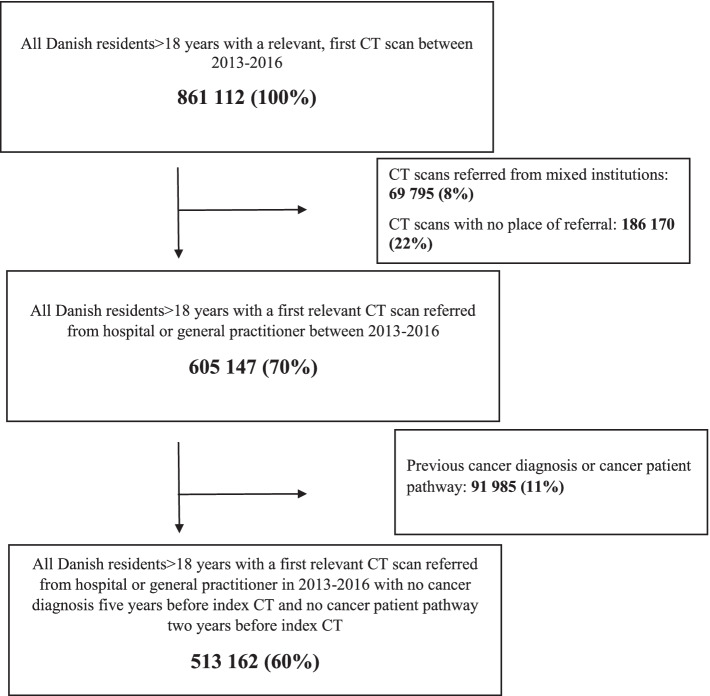
flow chart

As part of the initial work-up often includes a CT scan we decided to use an initial CT scan as the starting point of a potential identification of individuals who may be candidates for OS-CPP or NSSC-CPP. The study population thus consisted of citizens with a relevant CT scan between 1 January 2013 and 31 December 2016 (Fig. 1). Non-relevant CT scans were defined as CT scans that unlikely have been ordered with the purpose of detecting cancer onset, e.g. CT scans of shoulder, foot or ankle (Supplementary table 1). Individuals were included if they, at the time of their first CT scan (index CT), were 18 years or older and did not have another relevant CT scan within one year before their index CT. We excluded individuals that within five years before index CT were recorded with a cancer diagnosis in the Danish Cancer Registry (DCR) and individuals that within the last two years were registered as initiating a CPP. The study population was categorized according to the place of CT scan referral; 1) GP or 2) hospital. We excluded CTs with no indication of place of referral and a mixed group referred from institutions as prison, dentist etc. (Fig. 1).

### Defining outcomes

#### Use of CPPs

Type and total number of CPPs (OS-CPP and NSSC-CPP) measured six months after index CT. Data was obtained from The Danish National Patient Registry (NPR) [30]

#### Non-cancer diagnoses

Non-cancer diagnoses were selected based on a study identifying twenty broad diagnostic groups of chronic diagnoses, based on the International Classification of Diseases 10th Revision, as described in details elsewhere [31]. Diagnoses were measured six months after index CT and obtained from NPR [30]

#### Cancer diagnoses

Cancer diagnoses registered six months after index CT obtained from DCR [32]. We report the ten most common cancers as well as all cancers combined.

#### Total mortality and cancer-specific mortality

Total mortality and cancer specific mortality one year after index CT was obtained from the Danish Register of Causes of Death [33].

### Defining covariates

Sex, age, country of origin and Region were obtained at index date from the Population Register [34]. Age was categorized into 18-45, 46-59, 60-79 and ≥80 years. Country of origin categorized into Denmark, western and non-western countries. From the Danish Education Register [35], highest attained educational level was obtained and categorized: low, medium and high. Affiliation to the labour market was categorised into employed, unemployed/social benefits recipient, under education, or retired/other and obtained from the Danish Registry of Labour Market Affiliation [36]. Cohabitation status was obtained from the Danish Family Relations Database and categorized into married/cohabiting couple or single/living alone. Non-cancer morbidity was based on the selected 20 categories of non-cancer chronic disease [31]. Non-cancer morbidities were counted as the total number: 0,1,2,≥3 diagnosed within two years prior to and up to one month before the index CT. Non-cancer morbidities within a month before index CT was not included, as it may likely be related to the index CT.

### Statistical analysis

Analyses of baseline characteristics were performed with chi-squared test. For each of the above described outcomes we calculated the odds ratio (OR) of CT referred by GP versus CT referred by the hospital in a multivariable binary logistic regression model. We reported unadjusted and adjusted results where age was included as a continuous variable and sex, country of origin, Region, education, affiliation to work market and cohabitation status were included as categorical variables. Non-cancer morbidity was included as yes/no of each of the twenty comorbidities. SAS version 9.4 was used for statistical analysis.

## Results

### Individual baseline characteristics

We identified 228 522 CT scans referred by GPs and 284 640 referred by hospitals (CT-groups) (Table 1). Marked differences in baseline characteristics were seen for the variables non-cancer morbidity and Region. No marked differences were found in age, sex, country of origin, education, affiliation to work market, cohabitation status. Individuals with non-cancer morbidity were more likely to have a CT scan referred by hospital than GP. Individuals from the Capital Region and Region Zealand were more likely to have a CT scan referred by the hospital.

**Table 1:** Study population characteristics

**Characteristics**	All CT scans *(n=513 162)*	CT scans referred by GP *(n=228 522)*	CT scans referred by the hospital *(n=284 640)*	Missing values	*P*-value (χ^2^ test)
**Age, *n* (%)**				0	<.0001
18-45 years	113 376 (22.1)	48 591 (21.3)	64 785 (22.8)		
46-59 years	119 654 (23.3)	52 929 (23.2)	66 725 (23.4)		
60-79 years	94 850 (40.5)	94 850 (41.5)	112 792 (39.6)		
≥80 years	32 152 (14.1)	32 152 (14.1)	40 338 (14.2)		
**Gender, *n* (%)**				0	0.0016
Female	262 958 (51.2)	117 664 (51.5)	145 294 (51.0)		
Male	250 204 (48.8)	110 858 (48.5)	139 346 (49.0)		
**Country of origin, *n* (%)**				0	<.0001
Danish	467 068 (91.0)	209 222 (91.6)	257 846 (90.6)		
Western descent	15 935 (3.1)	6 805 (3.0)	9 130 (3.2)		
Non-western descent	30 159 (5.9)	12 495 (5.5)	17 664 (6.2)		
**Education, *n* (%)**				15 240	<.0001
Low	183 915 (36.9)	82 101 (36.9)	101 814 (36.9)		
Medium	203 898 (41.0)	91 752 (41.3)	112 146 (40.7)		
High	110 109 (22.1)	48 377 (21.8)	61 732 (22.4)		
**Affiliation to work market, *n* (%)**				33	<.0001
Employed	181 309 (35.3)	83 278 (36.4)	98 031 (34.4)		
Unemployed/social benefits	90 709 (17.7)	37 556 (16.4)	53 153 (18.7)		
Under education	13 536 (2.6)	5 257 (2.3)	8 279 (2.9)		
Retired/other	227 575 (44.4)	102 417 (44.8)	125 158 (44.0)		
**Cohabitation status, *n* (%)**				0	<.0001
Married or cohabiting	305 835 (59.6)	140 971 (61.7)	164 864 (57.9)		
Single/living alone	207 327 (40.4)	87 551 (38.3)	119 776 (42.1)		
**Region of residence, *n* (%)**				0	<.0001
Northern DK	70 310 (13.7)	36 444 (16.0)	33 866 (11.9)		
Central DK	106 933 (20.8)	53 746 (23.5)	53 187 (18.7)		
Southern DK	131 864 (25.7)	69 047 (30.2)	62 817 (22.1)		
Capital DK	142 697 (27.8)	49 112 (21.5)	93 585 (32.9)		
Zealand DK	61 358 (12.0)	20 173 (8.8)	41 185 (14.5)		
**Non-cancer morbidity, n (%)**				0	<.0001
No morbidities	360 620 (70.3)	175 955 (77.0)	184 665 (64.9)		
One morbidity	102 549 (20.0)	37 186 (16.3)	65 363 (23.0)		
Two morbidities	33 517 (6.5)	10 624 (4.7)	22 893 (8.0)		
Three or more morbidities	16 476 (3.2)	4 757 (2.1)	11 719 (4.1)		

### Type and number of CPPs

**Fig. 2:**
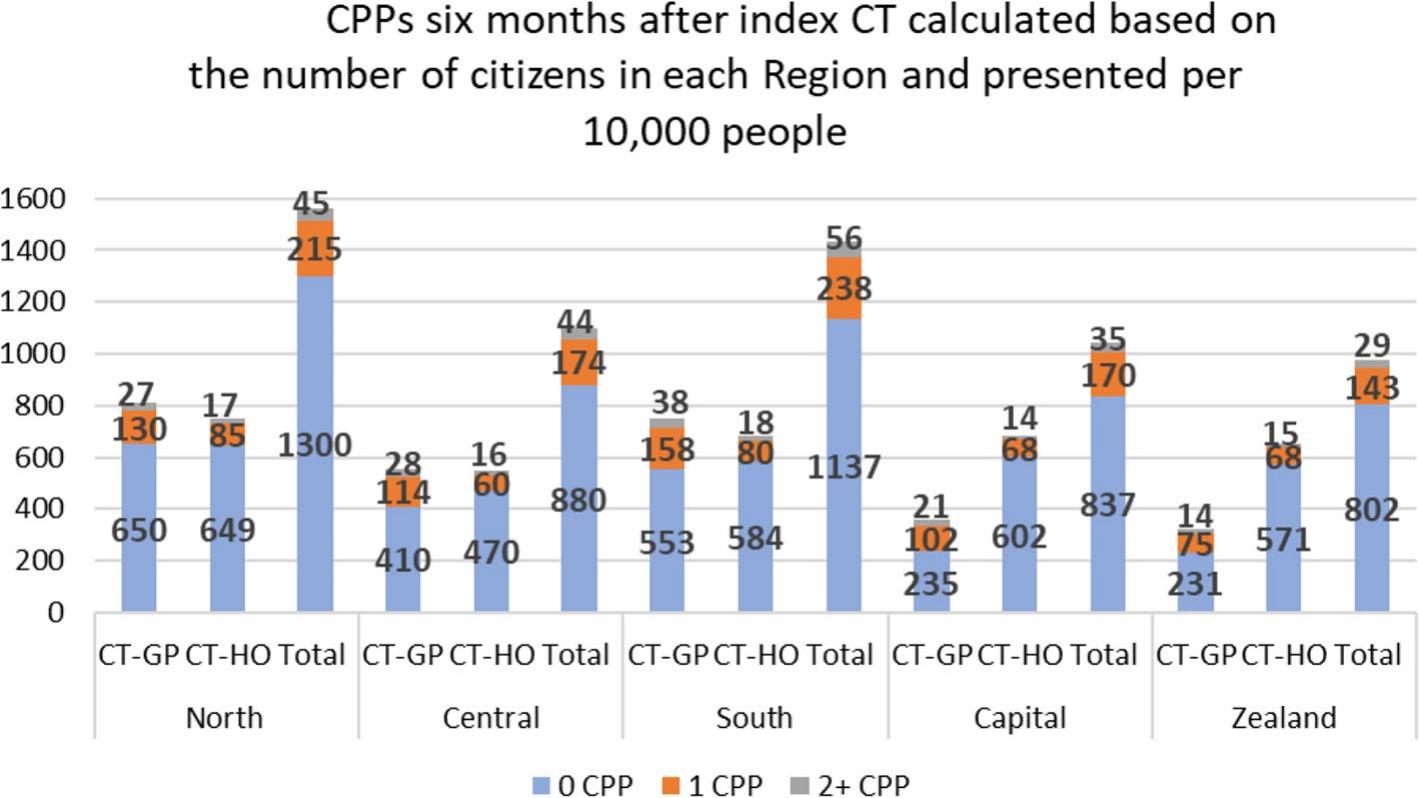
CPPs six months after index CT calculated based on the number of citizens in each Region and presented per 10,000 people

All the presented ORs in the results section are adjusted unless otherwise stated. Compared to CT scans referred by hospitals, CT scans referred by GPs had a four times higher odds (OR=4.06 [3.90 4.22]) of being followed by a NSSC-CPP and two times higher odds (OR=2.27 [2.23 2.30]) to be followed by an OS-CPP (Table 2). We found no statistically significant difference between total number of CPPs according to whether CT scans were referred by GP or hospital. Fig. 2 shows the number of CPPs divided by the total number of residents in each Region and CTs referred by GP and hospital, respectively.

**Table 2:** Type and number of CPPs

**All CT scans *(n=513 162)***	CT scans referred by GP *(n=228 522)*	CT scans referred by the hospital *(n=284 640)*	Crude OR (95%CI)	*P*-value	^a^Adjusted OR (95% CI)	*P*-value
**Type of first CPP 6 months after index CT**						
None	166 941 (73.1)	247 197 (86.9)	(ref)	-	(ref)	-
NSSC-CPP	9 899 (4.3)	3 866 (1.36)	3.79 (3.65 3.93)	<.0001	4.06 (3.90 4.22)	<.0001
OS-CPP	51 682 (22.6)	33 577 (11.8)	2.28 (2.25 2.31)	<.0001	2.27 (2.23 2.30)	<.0001
**Total number of CPPs 6 months after index CT**						
0 CPP	166 941 (73.1)	247 197 (86.9)	0.41 (0.41 0.42)	0.006	0.41 (0.40 0.42)	<.0001
1 CPP	50 261 (22.0)	30 658 (10.8)	(ref)	-	(ref)	-
2 CPP	9 387 (4.1)	5 580 (2.0)	1.03 (0.99 1.06)	0.161	1.00 (0.96 1.04)	0.970
3 CPP	1 595 (0.7)	1 038 (0.4)	0.94 (0.87 1.02)	0.110	0.90 (0.83 0.98)	0.015
4+CPP	338 (0.2)	167 (0.1)	0.67 (0.04 10.79)	0.778	1.16 (0.95 1.40)	0.141

^a^OR for CT-GP vs. CT-HO, adjusted for age, sex, country of origin, Region of residence, education, affiliation to work market, cohabitation status and non-cancer morbidity

### Cancers and non-cancers

35% of all CT scans resulted in a diagnosis of cancer or one of the selected non-cancer diagnoses. CT scans referred by GPs had a 12% higher odds of resulting in a cancer diagnosis than CT scans referred by hospitals (OR=1.12 [1.09 1.14]) (Table 3). Large variations were found within different cancer diagnoses: lung (OR=1.62 [1.55 1.69]), bladder (OR=1.80 [1.65 1.96]) brain/central nervous system (OR=1.40 [1.22 1.60]), pancreas (OR=1.50 [1.36 1.65]) and kidney (OR=1.37 [1.26 1.49]) were more often found in CT scans referred by GPs. For the non-cancer diagnoses, CT scans referred by GPs were more often followed by, stroke (OR=1.30 [1.27 1.33]), multiple sclerosis (OR=1.43 [1.19 1.72]) and dementia (OR=2.23 [2.15 2.32]).

**Table 3:** Diagnoses 6 months after index CT

**Diagnoses**	CT scans referred by GP *(n=228 522)*	CT scans referred by hospital *(n=284 640)*	Crude OR (95%CI)	*P*-value crude	^a^Adjusted OR (95% CI)	*P*-value adjusted
**All cancer and selected non-cancer diagnoses**	80 525 (35.2)	100 637 (35.4)	1.00 (0.98 1.01)	0.377	1.01 (1.00 1.03)	0.046
**All Cancers diagnoses**	20 926 (9.2)	22 567 (7.9)	1.17 (1.15 1.19)	<.0001	1.12 (1.09 1.14)	<.0001
Lung	5 031 (2.2)	3 831 (1.3)	1.65 (1.58 1.72)	<.0001	1.62 (1.55 1.69)	<.0001
Breast	2 846 (1.3)	4 301 (1.5)	0.82 (0.78 0.86)	<.0001	0.77 (0.73 0.81)	<.0001
Prostate	2 541 (1.1)	3 582 (1.3)	0.88 (0.84 0.93)	<.0001	0.85 (0.81 0.90)	<.0001
Colon	3 203 (1.4)	3 364 (1.2)	1.19 (1.13 1.25)	<.0001	1.11 (1.05 1.16)	0.0001
Melanoma of skin	778 (0.3)	943 (0.3)	1.03 (0.94 1.13)	0.573	1.02 (0.92 1.13)	0.7106
Bladder	1 445 (0.6)	977 (0.3)	1.85 (1.71 2.01)	<.0001	1.80 (1.65 1.96)	<.0001
Brain, central nervous system	477 (0.2)	421 (0.2)	1.42 (1.24 1.62)	<.0001	1.40 (1.22 1.60)	<.0001
Rectum and anus	1 189 (0.5)	1 691 (0.6)	0.88 (0.81 0.94)	0.0005	0.80 (0.74 0.86)	<.0001
Non-Hodgkin lymphoma	905 (0.4)	1 135 (0.4)	0.99 (0.91 1.08)	0.878	0.97 (0.88 1.06)	0.4406
Pancreas	1 006 (0.4)	792 (0.3)	1.59 (1.44 1.74)	<.0001	1.50 (1.36 1.65)	<.0001
Endometrium	492 (0.2)	567 (0.2)	1.08 (0.96 1.22)	0.2050	1.04 (0.92 1.18)	0.5476
Kidney	1 192 (0.5)	1 072 (0.4)	1.39 (1.28 1.51)	<.0001	1.37 (1.26 1.49)	<.0001
Ovary	498 (0.2)	508 (0.2)	1.22 (1.08 1.39)	0.0014	1.08 (0.95 1.23)	0.2154
Stomach	392 (0.2)	441 (0.2)	1.11 (0.97 1.27)	0.1403	1.03 (0.90 1.19)	0.6748
**Selected non-cancer diagnoses**	66 463 (29.1)	85 865 (30.2)	0.95 (0.94 0.96)	<.0001	0.97 (0.96 0.99)	<.0001
Coronary heart disease	7 519 (3.3)	15 978 (5.6)	0.572 (0.560.59)	<.0001	0.55 (0.54 0.57)	<.0001
Heart failure	3 309 (1.5)	6 762 (2.4)	0.60 (0.58 0.63)	<.0001	0.64 (0.61 0.67)	<.0001
Peripheral vascular disease	4 690 (2.1)	6 890 (2.4)	0.85 (0.81 0.88)	<.0001	0.84 (0.81 0.87)	<.0001
Chronic obstructive pulmonary disease	8 666 (3.8)	14 515 (5.1)	0.73 (0.71 0.75)	<.0001	0.79 (0.77 0.81)	<.0001
Diabetes	6 078 (2.7)	8 134 (2.9)	0.93 (0.90 0.96)	<.0001	0.96 (0.93 0.99)	0.022
Liver disease	2 353 (1.0)	3 181 (1.1)	0.92 (0.87 0.97)	0.0026	1.00 (0.94 1.05)	0.872
Thyroid disorders	3 317 (1.5)	4 073 (1.4)	1.02 (0.97 1.06)	0.539	1.03 (0.99 1.09)	0.1701
Kidney disease	3 618 (1.6)	4 643 (1.6)	0.97 (0.93 1.01)	0.176	0.96 (0.92 1.01)	0.096
Inflammatory bowel disease	2 325 (1.0)	2 817 (1.0)	1.03 (0.97 1.09)	0.321	1.06 (1.00 1.12)	0.042
Ulcer	1 939 (0.9)	2 930 (1.0)	0.82 (0.78 0.87)	<.0001	0.86 (0.81 0.92)	<.0001
Hemiplegia/stroke	19 427(8.5)	20 074 (7.1)	1.23 (1.20 1.25)	<.0001	1.30 (1.27 1.33)	<.0001
Parkinson disease	584 (0.3)	726 (0.3)	1.00 (0.90 1.12)	0.9999	1.02 (0.91 1.14)	0.791
Multiple sclerosis	236 (0.1)	233 (0.1)	1.26 (1.06 1.52)	0.011	1.43 (1.19 1.72)	0.0002
Epilepsy	2 063 (0.9)	3 182 (1.1)	0.81 (0.76 0.85)	<.0001	0.86 (0.81 0.91)	<.0001
Dementia	7 894 (3.5)	4 730 (1.7)	2.12 (2.04 2.20)	<.0001	2.23 (2.15 2.32)	<.0001
Osteoporosis	4 568 (2.0)	7 332 (2.6)	0.77 (0.74 0.80)	<.0001	0.70 (0.68 0.73)	<.0001
Rheumatoid and connective tissue disease	3 020 (1.3)	3 616 (1.3)	1.04 (0.99 1.09)	0.106	1.04 (0.99 1.09)	0.145
HIV/AIDS	50 (0.02)	66 (0.02)	0.94 (0.65 1.36)	0.758	1.05 (0.72 1.54)	0.786
Depression/ anxiety	4 940 (2.1)	6 381 (2.2)	0.96 (0.93 1.00)	0.053	1.06 (1.02 1.10)	0.0042
Psychotic diseases	679 (0.3)	1 427 (0.5)	0.59 (0.54 0.65)	<.0001	0.70 (0.63 0.77)	<.0001

^a^OR for cancer or selected non-cancer diagnoses in CT-GP vs. CT-HO, adjusted for age, sex, country of origin, Region of residence, education affiliation to work market, cohabitation status and non-cancer morbidity

### Cancers and non-cancers by Region

**Fig. 3:**
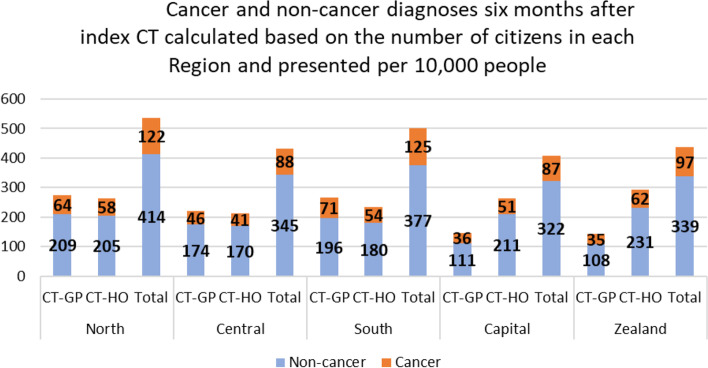
Cancer and non-cancer diagnoses six months after index CT calculated based on the number of citizens in each Region and presented per 10,000 people

In Region Zealand, 34% of CT scans were followed by a non-cancer diagnosis, 10% by cancer and 40% either by a non-cancer or a cancer diagnosis (Table 4). Reported in the same order, 1) non-cancer, 2) cancer and 3) non-cancer or cancer, the percentages were 27%,8%,32% in Region North, 31%,8%,37% in Central, 26%,9%,32% in South and 31%,8%,36% in Capital Region, respectively. CT scans referred by GPs were more likely to be followed by a cancer diagnosis than CT scans referred by hospitals in Regions South (OR=1.10 [1.06 1.15]), Zealand (OR=1.15 [1.09 1.22]) and Capital (OR=1.26 [1.21 1.31]),. The same tendency was observed in the Central (OR=1.05 [1.00-1.10]) while there was no difference in the North Region (OR=0.97 [0.92 1.03]. Fig. 3 provides the cancer and non-cancer diagnoses calculated based on the number of citizens in each Region and presented by 10,000 people.

**Table 4:** Non-cancer and cancer diagnoses 6 months after index CT based on CT scans referred by GP (CT-GP) and hospital (CT-HO) in each Region and between Regions

**Diagnoses 6 months after index CT scan by CT scans referred by GP and hospital in each Region Total=513 162, *n*(%)**					Diagnoses 6 months after index CT scan by Region Total= 513 162, *n*(%)		
**Regions and CT-groups**		**Non-cancer**	**Cancer**	**Non-cancer or cancer**	**Non-cancer**	**Cancer**	**Non-cancer or cancer**
**All Regions**	CT-GP *n*=228 522	66 463 (29.1)	20 926 (9.2)	80 525 (35.2)	152 328 (29.7)	43 493 (8.5)	181 162 (35.3)
	CT-HO *n*=284 640	85 865 (30.2)	22 567 (7.9)	100 637 (35.4)			
	Adjusted OR (95%CI)	^a^0.97 (0.96 0.99)	^b^1.12 (1.09 1.14)	^a^1.01 (1.00 1.03)			
	*P* -value	<.0001	<.0001	0.046			
**Zealand**^**a**^	CT-GP *n*=20 173	6 803 (33.7)	2 178 (10.8)	8 183 (40.6)	21 361 (34.3)	6 088 (9.8)	25 114 (40.4)
	CT-HO *n*=41 185	14 559 (35.4)	3 910 (9.5)	16 931 (41.1)			
	Adjusted OR (95%CI)	^a^1.00 (0.96 1.04)	^b^1.15 (1.09 1.22)	^a^1.06 (1.02 1.10)			
	*P* -value	0.979	<.0001	0.0015			
**North**^**b**^	CT-GP *n*=36 444	9 439 (25.9)	2 868 (7.9)	11 362 (31.2)	18 661 (26.5)	5 496 (7.8)	22 313 (31.7)
	CT-HO *n*=33 866	9 222 (27.2)	2628 (7.8)	10 951 (32.3)			
	Adjusted OR (95%CI)	^a^0.94 (0.90 0.97)	^b^0.97 (0.92 1.03)	^a^0.95 (0.91 0.98)			
	*P* -value	0.0003	0.332	0.0013			
**Central**^**b**^	CT-GP *n*=53 746	16 957 (31.6)	4 519 (8.4)	19 981 (37.2)	33 553 (31.2)	8 538 (8.0)	39 277 (36.7)
	CT-HO *n*=53 187	16 596 (31.2)	4 019 (7.6)	19 296 (36.3)			
	Adjusted OR (95%CI)	^a^0.99 (0.96 1.01)	^b^1.05 (1.00 1.10)	^a^1.01 (0.98 1.03)			
	*P* -value	0.348	0.049	0.686			
**South**^**b**^	CT-GP *n*=69 047	18 091 (26.2)	6 503 (9.4)	22 660 (32.8)	34 725 (26.3)	11 508 (8.7)	42 728 (32.4)
	CT-HO *n* =62 817	16 634 (26.5)	5 005 (8.0)	20 068 (32.0)			
	Adjusted OR (95%CI)	^a^0.95 (0.93 0.98)	^b^1.10 (1.06 1.15)	^a^1.00 (0.97 1.02)			
	*P* -value	0.0004	<.0001	0.916			
**Capital**^**c**^	CT-GP *n* =49 112	15 173 (30.9)	4 858 (9.9)	18 339 (37.3)	44 027 (30.9)	11 863 (8.3)	51 730 (36.3)
	CT-HO *n* =93 585	28 854 (30.8)	7 005 (7.5)	33 391 (36.0)			
	Adjusted OR (95%)	^a^0.99 (0.97 1.02)	^b^1.26 (1.21 1.31)	^a^1.06 (1.03 1.08)			
	*P*-value	0.474	<.0001	<.0001			

a=GPs responsible for the initial CT scan before NSSC-CPP

b=intraregional differences regarding responsibility of initial CT scan

c=hospital responsible for the initial CT scan before NSSC-CPP

^a^OR for CT-GP vs. CT-HO, adjusted for age, sex, country of origin, Region of residence, education, affiliation to work market, cohabitation status

^b^OR for CT-GP vs. CT-HO, adjusted for age, sex, country of origin, Region of residence, education, affiliation to work market, cohabitation status and non-cancer morbidity

### Total and cancer-specific mortality

Individuals with a CT scan referred by GPs had a 12% reduced odds (OR=0.88 [0.86 0.90]) of dying (total mortality) compared to individuals with a CT scan referred by hospital (Table 5). The cancer-specific mortality was 3% when CT scan was referred by GPs and 2% when referred by hospital, resulting in a 26% higher odds of dying from cancer among individuals with a CT scan referred by GPs (OR=1.26 [1.21 1.30]).

**Table 5:** Total mortality and cancer-specific mortality 1 year after index CT scan based on CT scans referred by GP (CT-GP) and hospital (CT-HO) in each Region and between Regions

**Mortality 1 year after index CT scan by CT scans referred by GP and hospital in each Region Total=513 162**				Total mortality one year after index CT scan by Region Total= 513 162	
**Regions and CT-groups**		**Total mortality *n*(%)**	**Cancer-specific mortality *n*(%)**	**Total mortality *n*(%)**	**Cancer specific mortality *n*(%)**
**All Regions**	CT-GP *n* =228 522	16 297 (7.13)	6 804 (3.0)	41 077 (8.0)	13 478 (2.6)
	CT-HO *n* =284 640	24 780 (8.7)	6 674 (2.3)		
	^a^Adjusted OR (95 %)	0.88 (0.86 0.90)	1.26 (1.21 1.30)		
	*P*-value	<.0001	<.0001		
**Zealand**^**a**^	CT-GP *n* =20 173	1 660 (8.2)	754 (3.7)	6 261 (10.2)	2 021 (3.3)
	CT-HO *n* =41 185	4 601 (11.2)	1 267 (3.1)		
	^a^Adjusted OR (95 %)	0.85 (0.79 0.90)	1.28 (1.16 1.41)		
	*P* -value	<.0001	<.0001		
**North**^**b**^	CT-GP *n* =36 444	2 602 (7.1)	954 (2.6)	11 507 (8.1)	1 823 (2.6)
	CT-HO *n* =33 866	3 147 (9.3)	869 (2.6)		
	^a^Adjusted OR (95%)	0.78 (0.73 0.83)	1.00 (0.91 1.10)		
	*P* -value	<.0001	0.992		
**Central**^**b**^	CT-GP *n* =53 746	4 025 (7.5)	1 409 (2.6)	8 411 (7.9)	2 569 (2.4)
	CT-HO *n* =53 187	4 386 (8.3)	1 160 (2.2)		
	^a^Adjusted OR (95 %)	0.92 (0.87 0.96)	1.15 (1.06 1.25)		
	*P* -value	0.0006	0.0006		
**South**^**b**^	CT-GP *n* =69 047	4 348 (6.3)	2 080 (3.0)	9 149 (6.9)	3 496 (2.7)
	CT-HO *n* =62 817	4 801 (7.6)	1 416 (2.3)		
	^a^Adjusted OR (95 %)	0.85 (0.81 0.89)	1.27 (1.18 1.36)		
	*P* -value	<.0001	<.0001		
**Capital**^**c**^	CT-GP *n* =49 112	3 662 (7.5)	1 607 (3.3)	5 749 (8.2)	3 569 (2.5)
	CT-HO *n* =93 585	7 845 (8.4)	1 962 (2.1)		
	^a^Adjusted OR (95 %)	0.96 (0.91 1.00)	1.51 (1.40 1.62)		
	*P* -value	0.048	<.0001		

a=GPs responsible for the initial CT scan before NSSC-CPP

b=intraregional differences regarding responsibility of initial CT scan

c=hospital responsible for the initial CT scan before NSSC-CPP

^a^OR for CT-GP vs. CT-HO, adjusted for age, sex, country of origin, Region of residence, education, affiliation to work market, cohabitation status and non-cancer morbidity

### Total and cancer-specific mortality by Region

Mortality was similar across the five regions, with total mortality of 8-10% and cancer-specific mortality of 3%. In all Regions, CT scans referred by GPs were associated with reduced odds of total mortality: North (OR=0.78 [0.73 0.83]), Central (OR=0.92 [0.87 0.96]), South (OR=0.85 [0.81 0.89]), Capital (OR=0.96 [0.91 1.00]) and Zealand (OR=0.85 [0.79 0.90]). Regarding cancer-specific mortality, in Central (OR=1.15 [1.06 1.25]), South (1.27 [1.18 1.36]), Zealand (1.28 [1.16 1.41]) and the Capital Region (1.51 [1.40 1.62]) CT scans referred by GPs where associated with an increased odds of cancer-specific mortality compared to CT scans referred by hospitals. In Region North, no difference was found (OR=1.00 [0.91 1.10]).

## Discussion

### Main findings

CT scans referred by GPs were more likely to be followed by an OS-CPP or a NSSC-CPP than CT scans referred by hospitals. Besides in Region North, CT scans referred by GPs were more likely to be followed by a cancer diagnosis and cancer-specific mortality. Individuals with a CT scan referred by GPs were less likely to die within one year from index CT scan (total mortality) but more likely to die from cancer (cancer-specific mortality). We found no clear pattern between high use of CT scans and CPPs and the following number of diagnostic outcomes and mortality.

### Strengths and limitations

The use of national register data made it possible to include a large sample and to adjust for a number of potential confounders which strengthen the credibility of this study. Also, the use of population-based register data, collected independently of study hypothesis, eliminated the risk of selection and information bias. Our exclusion criteria enabled us to investigate a naïve population regarding CPPs and cancer diagnoses and thereby avoiding that CT scans were parts of follow-up after e.g. cancer treatment.

An important challenge and limitation of this study relates to the exposure measurement. We chose place of CT referral (GP or hospital), as we in a previous study found differences between and within regions in place of initial CT scan [19]. However, CT scans ordered with the suspicion of cancer are not labelled ‘suspicion of cancer’ in the registers. Therefore, we had to include all CT scans and thereby describe a broader diagnostic pattern. Still, as we wanted to describe and compare the outcomes of the initial diagnostic work performed by GPs and hospitals, this was, in our opinion, the best suitable design.

A high proportion of total CT scans were excluded due to missing referral information. When examining, this excluded group only, they were less likely to be followed by a CPP than individuals referred by both GP and hospital referred CT scans.

### Interpretations of results and comparison with other studies

We found that CT scans referred by GPs were more likely to be followed by a NSSC-CPP and an OS-CPP. This is not surprising as, according to the Danish Health Authority, 70% of NSSC-CPPs and 53% of OS-CPPs are initiated after referral from GPs [37, 38]. We found no difference between the CT-groups and the total numbers of CPPs. A Danish registry-study, found that 6% of patients going through an initial OS-CPP, without receiving a diagnosis of cancer, were re-referred to one additional or more CPPs within a six month period [39]. In our study, this number was 22%. These results should be compared with caution, as we did also include the NSSC-CPP. As only few local studies have looked into the diagnostic outcomes of CPPs, it is uncertain whether more CPPs within a short period of time are more costly to society and cause more benefits than harms to the individual.

We found that CT scans referred by GPs were, with large regional variations, more likely to be followed by a cancer diagnosis and cancer-specific mortality. This might indicate the importance of easy access to diagnostic imaging for GP’s as they play an essential role in initial cancer diagnostics. Probably it also reflects a broader use of CT scans within patients in the hospitals who represent a selected population with morbidity. From these results, we cannot conclude that patients starting their initial cancer diagnostics at GPs are worse off than patients referred from hospitals, but that they are in general more associated to a cancer diagnosis and thereby cancer-specific mortality. CT scans referred from hospitals were follow by a higher total mortality. This again might be due to hospitals more likely referring trauma patients and patients with other severe conditions to a CT scan than GPs. Regional variations may be interpreted as the Regions’ use of CT scans between GPs and hospitals are organised differently. For example, in Region Zealand, CT scans referred by GPs had a 15% greater odds of being followed by a cancer diagnosis than CT scans referred by hospitals. This result correspond to our previous publication, showing that in Region Zealand, the GPs are responsible for the initial diagnostic work up in patients with non-specific symptoms that could be cancer [19]. However, in the same study, the hospitals in the Capital region are responsible for the initial CT scan, but in the present study CT scans referred by GPs had a 26% greater risk of being followed by a cancer diagnosis than CT scans referred by hospitals. This indicates that other factors might be more important than the regional diagnostic infrastructure and that several routes to diagnosis exist. We found no clear association between high use of CT scans and CPPs in some Regions and the following pattern of diagnoses and mortality, e.g. Region Zealand had the lowest use of CT scans and CPPs, and the highest proportion of non-cancer and cancer diagnoses, as well as the highest total and cancer-specific mortality (all outcomes being statistically significant when adjusted for potential confounders in a multiple logistic regression model). This could be interpreted as Region Zealand refers too few patients to CT scans and CPPs in both primary and secondary health care, as the ones referred to CT scans have a greater risk of diagnoses and mortality than other Regions. Opposite, the Capital Region had a similar low use of CT scans and CPPs, but a lower proportion of following cancer diagnoses and a similar total and cancer-specific mortality as the other Regions. This indicates that neither the proportion of CT scans and CPPs nor the different modality (GP or hospital responsible for the initial diagnostic work up) can explain the diagnostic outcomes and mortality. Possible explanations might be population based: health behaviour such as smoking or alcohol consumption, or social factors as loneliness and network support – or other unmeasurable confounders. Qualitative and quantitative research is needed to explore and to estimate which factors that might explain the regional differences in diagnoses and mortality found in this study.

### Implications

No studies have investigated the use of CPPs and diagnostic outcomes after the initial cancer diagnostic, even though these outcomes are crucial when evaluating the benefits and harms of cancer diagnostic in Denmark. We acknowledge, that this study provides an overarching description, indicating the need for studies aiming to answer the question - which diagnostic model is the most beneficial and least harmful? This, however, was beyond the scope of the present study. A challenge, to such future studies lies in the registration of the diagnostic work up in general practice. Still, our results indicate that Regions use CT scans differently in cancer diagnostic, and that Regions with a large use of CT scans and CPPs do not necessarily have a corresponding low or high number of cancer diagnoses or cancer-specific mortality. This is important knowledge to the Danish Health Authorities, as they are currently measuring the quality of the NSSC-CPP based on the number of completed NSSC-CPPs. Our study indicates that this is not a sufficient quality indicator, and that more patient prognostic outcomes should be included when evaluating the quality of cancer diagnostics. Further, in 2016, Norway and Sweden implemented NSSC-CPPs based on the Danish model [6, 10]. As we have not been able to identify studies investigating their potential organisational differences, results from our study might be relevant in an international context.

## Conclusion

We found that Regions have organised their use of CT scans in cancer diagnostics differently between GPs and hospitals. CT scans referred by GPs were more likely to be followed by a CPP, a cancer diagnosis and cancer-specific mortality. This may be due to differences in patient populations in hospitals and GPs. No obvious association between number of CT scans and CPPs and the following prognostic outcomes were found. These results are important when evaluating the quality of initial cancer diagnostic in Demark.

## Supplementary Information

**Additional file 1.** **Supplementary table 1.** Non-relevant CT scans were defined as CT scans that unlikely have been ordered with the purpose of detecting cancer onset and were excluded from our study.

## Data Availability

The data that support the findings of this study are available from Denmark Statistic (https://www.dst.dk/da/) and the Danish Cancer Registry (https://sundhedsdatastyrelsen.dk/da/registre-og-services/om-de-nationale-sundhedsregistre/sygedomme-laegemidler-og-behandlinger/cancerregisteret) but restrictions apply to the availability of these data, which were used under license for the current study, and so are not publicly available. Please contact the corresponding author for data requests. All methods were carried out in accordance with relevant guidelines and regulations.
