# Supplementary material for: Use and diagnostic outcomes of cancer patient pathways in Denmark – is the place of initial diagnostic work-up an important factor?
Source: BMC Health Serv Res. 2022 Jan 31;22:130. doi: 10.1186/s12913-022-07545-x (PMC8802524; doi:10.1186/s12913-022-07545-x)
Supplement: Supplementary file 1 — Additional file 1. Supplementary table 1. Non-relevant CT scans were defined as CT scans that unlikely have been ordered with the purpose of detecting cancer onset and were excluded from our study. [file 12913_2022_7545_MOESM1_ESM.docx]

**Supplementary table 1**

| Danish | English |
| --- | --- |
|  |  |
| UXCG50 CT-skanning af fod | UXCG50 CT scan of food |
| UXCA90 CT-skanning af traumept. (hoved, thorax, abdomen og bækken) | UXCA90 trauma CT scan (head, thorax, abdomen and pelvis) |
| UXCG25 CT-skanning af knæ | UXCG25 CT scan of knee |
| UXCG40 CT-skanning af fodled | UXCG40 CT scan of ankle |
| UXCF15 CT-skanning af skulder | UXCF15 CT scan of shoulder |
| UXCF40 CT-skanning af håndled | UXCF40 CT scan of wrist |
| UXCF25 CT-skanning af albue | UXCF25 CT scan of elbow |
| UXCF50 CT-skanning af hånd | UXCF50 CT scan of hand |
| UXCG80 CT-osteodensitometri af hofte | UXCG80 CT- osteodensitometry of hip |
| UXCA60 CT-skanning af mandiblen | UXCA60 CT scan of mandible |
| UXCF30 CT-skanning af underarm | UXCF30 CT scan of forearm |
| UXCA65 CT-skanning af kæbeled | UXCA65 CT scan of jaw joint |
| UXCA55 CT-skanning af tænder | UXCA55 CT scan of teeth |

Non-relevant CT scans were defined as CT scans that unlikely have been ordered with the purpose of detecting cancer onset and were excluded from our study. Full list below.
